# Extraction Optimization of Polyphenols from Waste Kiwi Fruit Seeds (*Actinidia chinensis* Planch.) and Evaluation of Its Antioxidant and Anti-Inflammatory Properties

**DOI:** 10.3390/molecules21070832

**Published:** 2016-06-25

**Authors:** Jianjun Deng, Qingqing Liu, Chao Zhang, Wei Cao, Daidi Fan, Haixia Yang

**Affiliations:** 1 Shaanxi Key Laboratory of Degradable Biomedical Materials, Department of Food Science and Engineering, College of Chemical Engineering, Northwest University, Xi’an 710069, China; dengjianjun@nwu.edu.cn (J.D.); qingqing1874379@163.com (Q.L.); caowei@nwu.edu.cn (W.C.); 2 Department of Nutrition and Food Safety, School of Public Health, Xi’an Jiao Tong University, Xi’an 710061, China; yyyyyy_214@163.com

**Keywords:** kiwi fruit seeds, polyphenols, antioxidation, anti-inflammatory

## Abstract

Kiwi fruit (*Actinidia chinensis* Planch.) seeds, present as a by-product in the food and pharmaceutical industries, remain underutilized. In this study the extraction conditions for the maximum recovery of total phenolic content (TPC) with high DPPH scavenging capacities (DPPHsc) were analyzed for kiwi fruit seed polyphenols (KSP) by response surface methodology. The optimal conditions for the highest yield of TPC (53.73 mg GAE/g DW) with 63.25% DPPHsc was found by using an extraction time of 79.65 min with an eluent containing 59.45% acetone at 38.35 °C and a 1:11.52 (*w*/*v*) solid/liquid ratio. Compared with butyl hydroxy toluene (BHT), a synthetic antioxidant, the extracted KSP showed higher DPPHsc and ferric reducing antioxidant power, but was less efficient than grape seed polyphenols extracted under the same optimum conditions. We also showed that the extracted KSP exhibited strong anti-inflammatory activities by suppressing the secretion of pro-inflammatory cytokines like interleukin-1β (IL-1β) and tumor necrosis factor-alpha (TNF-α) in lipopolysaccharides (LPS)-induced RAW 264.7 cells. High performance liquid chromatography-electrochemical detector (HPLC-ECD) analysis of the extracted KSP under optimized conditions revealed that the extract was mainly composed of five polyphenolic compounds. Our work showed the development of an optimal extraction process of the KSP, which presented excellent antioxidant and anti-inflammatory activities, indicating that kiwi fruit seeds may further be utilized as a potential source of natural biological active compounds.

## 1. Introduction

Polyphenolic compounds are most abundantly present in natural bioactive compounds. These compounds commonly found in fruits, vegetables, and cereals are known for their medicinal properties [1,2,3]. They exhibit a wide range of pharmacological properties such as antimicrobial and anti-inflammatory activities [4,5], and the widely accepted mechanism behind these properties involves the reduction of oxidative stress by scavenging free radicals [6].

Industrial processing of fruits and vegetables generates a large amount of waste and these by-products could be managed and utilized more efficiently. These by-products are, in fact, rich in bioactive components from different fruit seeds, such as grape seeds [7] and fenugreek seeds [8]. By-products from the food and cosmetic industries are now considered as new sources of polyphenolic compounds, and therefore the effective utilization of these waste materials has recently generated increasing amount of interest among scientists [9]. Kiwi fruit (*Actinidia chinensis* Planch.), also known as the Chinese gooseberry, is an edible berry that originated from the central and southern regions of China. Kiwi fruit seeds represent 33–46 g/kg of the edible part of a kiwi fruit and are generated as solid wastes from food industries [9]. Kiwi fruit seed oil and proteins have been successfully extracted and considered as a new nutrient suitable for applications in food, health, and cosmetic industries [10,11]. However, the biological activities of polyphenols extracted from kiwi fruit seeds (KSP) still remains unknown. Therefore, it is worth optimizing the extraction conditions and investigating the biological activities of the KSP.

Hence, the general objective of this work was aimed at optimizing the extraction process of polyphenols from kiwi fruit seeds to ensure a maximum recovery of polyphenols with a high antioxidant activity. The major polyphenolic compounds were identified using high performance liquid chromatography-electrochemical detector (HPLC-ECD). Additionally, antioxidant and anti-inflammatory properties of KSP were determined in order to evaluate its potential as a food supplement.

## 2. Results and Discussion

### 2.1. Fitting Model

The extraction process is an important step for isolating and identifying polyphenols. To optimize the parameters of the extraction of polyphenols from kiwi fruit seeds, a preliminary single factor test was performed to narrow down the ranges of the independent variables (extraction time, acetone concentration, temperature, and liquid/solid ratio) by response surface methodology (RSM) (Table 1). Next, KSP was extracted following 31 selected combinations consisting of the four variables as per the experimental design. The experimental values for the total phenolic content (TPC) and DPPH scavenging capacities (DPPHsc) were not statistically different when compared to the predicted values (*p* > 0.05), indicating a satisfactory model (Table 2). The experimental values of both response variables were employed in a multiple regression analysis performed using a response surface analysis to fit the second-order polynomial equations. Table 3 presents the predicted model. ANOVA was used to examine the statistical significance of the model. The R^2^ values for the regression model of TPC and DPPHsc were 96.54% and 94.20%, respectively, indicating that the model is a reasonable fit to the experimental data. Earlier studies have reported R^2^ values ranging from 95% to 99.86% [1,12,13]. The lack of fit testing was performed to verify the adequacy of the fit. Both the lack of fits of 0.1764 and 0.3545 did not show inadequacy of the model with regard to TPC and DPPHsc (*p* > 0.05), thus indicating the suitability of the models to predict the variation accurately.

### 2.2. Influence of Extraction Parameters on TPC

**Figure 1:**
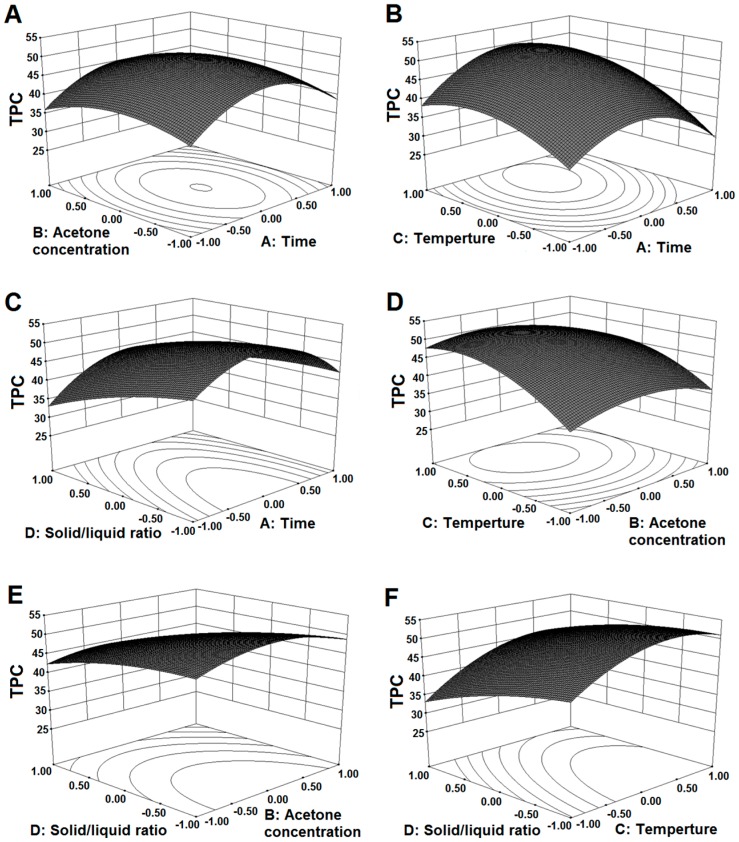
Response surface plots for the effect of acetone concentration and time (**A**); temperature and time (**B**); solid/liquid ratio and time (**C**); temperature and acetone concentration (**D**); solid/liquid ratio and acetone concentration (**E**) and solid/liquid ratio and temperature (**F**) on the TPC (mg GAE/g DW) of KSP.

The mean experimental TPC obtained from the triplicate mean values varied from 11.11 to 55.10 mg GAE/g DW, which is higher than that from *Mangifera pajang* Kosterm peel [13] and neem (*Azadirachta indica*) leaves [14]. Using the fitted model mentioned above, three-dimensional (3D) response surface plots were obtained to spot the region of optimal TPC extraction, by simultaneously keeping the two independent variables (Figure 1). Extraction time, acetone concentration, temperature, and solid/liquid ratio showed greater influence on KSP extraction. Regarding the extraction time (*X*_1_), we found a significant negative quadratic effect of *X*_1_ (*p* < 0.05) for the TPC extraction, indicating that there was a maximum yield point in the KSP extraction at an extraction time of 80 min (Figure 1A,B). KSP yield starts to decrease above this point. Further increase in the extraction time degraded the phenolic compounds due to interference in compound stability caused by chemical and enzymatic degradation or reaction with other components, thus reducing the extraction efficiency [15]. The acetone concentration (*X*_2_) was another important parameter in the extraction procedure. Similar to the extraction time, the quadratic effect of the acetone concentration on the KSP extraction was significant (*p* < 0.05), as shown in Figure 1A,D. The TPC increased with increasing acetone concentration up to 60%, followed by a reduction until reaching a minimum value at 100% acetone concentration. The results were in agreement with that reported by Uma et al. [15] but not with the results from Gong et al. [1]. A remarkable drop in the TPC at 100% acetone concentration revealed low polyphenol solubility in absolute solvent. This observation indicated that the extraction of phenolic compounds largely depended on the polarity of the solvents and compounds; a single solvent might not be effective for isolating a bioactive compound. Moreover, the extraction temperature (*X*_3_) was another main parameter in the extraction procedure. As shown in Figure 1B,D, effects of linear, quadratic, and interaction with time are significant on the TPC. With the increased temperature, the TPC showed a linear effect below 40 °C. However, excessively high temperatures did not increase the extraction yield. We avoided increasing the extraction temperature to avoid the heat degradation of the polyphenols. Apart from enhancing the solubility of polyphenolic compounds, mild heating might also weaken the membrane structure of plant cells and soften plant tissues [16]. Finally, we found that the solid/liquid ratio (*X*_4_) played a significant role on the TPC. As shown in Figure 1E,F, there was a decline in the TPC content with an increasing solid/liquid ratio. Our results were consistent with the reports of Gan and Latif [3], indicating that the solid/liquid ratio can significantly affect the yield of polyphenols.

### 2.3. Influ ence of Extraction Parameters on DPPHsc

**Figure 2:**
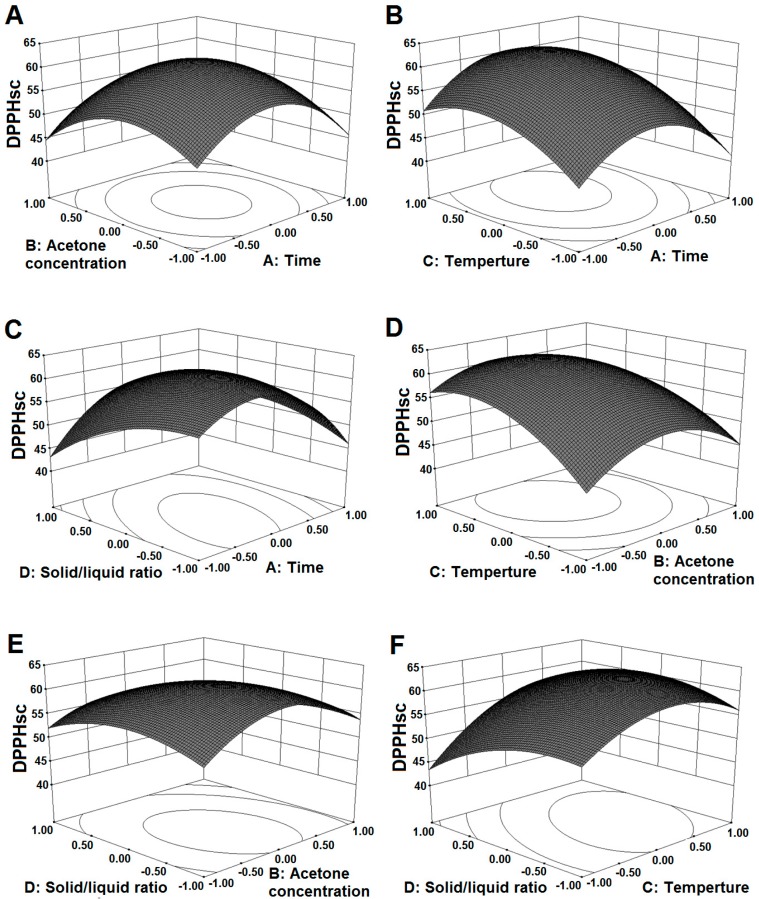
Response surface plots for the effect of acetone concentration and time (**A**); temperature and time (**B**); solid/liquid ratio and time (**C**); temperature and acetone concentration (**D**); solid/liquid ratio and acetone concentration (**E**) and solid/liquid ratio and temperature (**F**) on the DPPH radical scavenging activity of KSP.

The DPPH scavenging activity was determined to evaluate the antioxidant activity of the extracted KSP. The ANOVA results of the second-order polynomial regression equation indicated that the effects of the temperature and solid/liquid ratio were significant (*p* < 0.0001 or *p* < 0.05) in a linear effect (*X*_3_, *X*_4_). All the quadratic effects (*X*_1_^2^, *X_2_^2^*, *X*_3_^2^, *X*_4_^2^) and interactive effects between the extraction time and the temperature (*X*_1_*X*_4_), and the extraction time and the solid/liquid ratio (*X*_3_*X*_4_) were also significant (*p* < 0.05), respectively. Table 3 presents the predicted model obtained for *Y*_2_. Figure 2 shows the significant quadratic effect of the extraction time on DPPHsc and depicts a higher DPPHsc in the region where the extraction time was about 80 min (Figure 2A,B). This trend was similar to the findings of Prasad et al. [13]. The acetone concentration displayed a similar effect with the extraction time on DPPHsc of KSP (Figure 2A,D). Singh et al. have also shown that radical scavenging ability increases with increasing solvent fraction, but with further increase, the percentage of inhibition of DPPH radicals starts to decline [17]. Additionally, Figure 2B,D clearly show that the temperature caused a linear increase in the DPPHsc, indicating the presence of highly thermostable antioxidants in the KSP extracts. This result was in accordance with the findings reported by Liyana-Parthirana and Shahidi on wheat [18]. Figure 2E,F depict the influence of the solid/liquid ratio on DPPHsc. A linear decrease of DPPHsc with increasing solid/liquid ratio might result from dilution of the extract solution.

### 2.4. Optimization of Extraction Conditions for TPC and DPPHsc

Comparing the 3D surface plots of the two responses, we found that the extraction conditions had similar effects on TPC and DPPHsc. Our results showed that the polyphenolic compounds probably played an important role in the DPPH scavenging capacity of KSP, which is consistent with previous reports [19]. To obtain the optimum conditions for KSP extraction, two single response variables were separately used to optimize the progress respectively. For the TPC, the optimum conditions were as follows: extraction time of 79.65 min, acetone concentration of 59.45%, extraction temperature of 38.35 °C, and solid/liquid ratio of 1:11.52 (*w*/*v*). The optimum conditions for DPPHsc were as follows: extraction time of 79.85 min, acetone concentration of 59.95%, extraction temperature of 38.20 °C, and solid/liquid ratio of 1:9.30 (*w*/*v*). We did not find any significant difference by comparing the two optimum conditions. The two experimental values according to the optimum conditions for the two responses were determined to validate the adequacy of the response surface models. We obtained a high fit degree between the experimental values (53.73 mg GAE/g seed for TPC and 63.25% for DPPHsc) and the predicted values (53.15 mg GAE/g seed for TPC and 62.77% for DPPHsc) (Table 2). However, our results are not consistent with the results reported by Prasad et al. on *Mangifera pajang* Kosterm. peels [13], which may be due to the presence of different polyphenolic compounds in plant materials. Moreover, correlation between TPC and DPPHsc at the optimum conditions indicated that TPC was significantly in positive correlation (R^2^ = 0.92, *p* < 0.01) with DPPHsc, indicating TPC could be used as an important indicator of antioxidant activity evaluated by DPPHsc.

### 2.5. Antioxidant Activities of KSP

**Figure 3:**
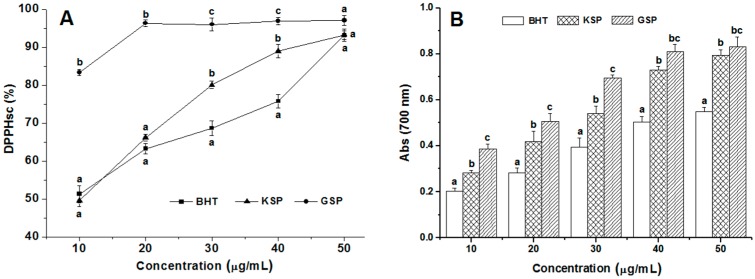
DPPH free radical scavenging (**A**) and ferric reducing antioxidant power (**B**) effects of the KSP in comparison with grape seed polyphenols (GSP) and butyl hydroxy toluene (BHT). Bar with the different letters represent values that significantly different according to the Duncan’s multiple range test (*p* < 0.05). Vertical bars represent the standard error from the means of three separate tests.

To compare the antioxidant activity of KSP with other antioxidants, we determined the DPPHsc and the ferric reducing antioxidant power (FRAP) at different concentrations (10–50 μg/mL) using butylated hydroxytoluene (BHT) and grape seed polyphenols (GSP) as positive controls. As shown in Figure 3A, the DPPHsc of KSP was compared with BHT and GSP, and revealed that the scavenging effects of the studied samples on the DPPH radical decreased in the following order: GSP > KSP > BHT. All the samples exhibited similar activities at high concentration. It is worth noticing that KSP exhibited an effective radical scavenging activity, significantly stronger (*p* < 0.05) than that of BHT at 30 μg/mL and 40 μg/mL concentration. The maximum DPPHsc of KSP was 93.17% at 50 μg/mL. This result was mainly attributed to the higher phenolic content of KSP, which is lower than that of the GSP samples obtained under the same extraction optimal conditions.

The FRAP assay is often used to evaluate the ability of natural antioxidants to donate an electron or hydrogen [20]. Figure 3B illustrates the FRAP of KSP and reference antioxidants at various levels. As the concentration of the samples increased, the FRAP activities increased (*p* < 0.05). The FRAP of KSP was significantly higher than that of BHT at different concentrations, but lower than that of GSP. The antioxidant activity of the phenolic acids depends on the number and position of the hydroxyl groups bound to the aromatic ring [21]. Our results were generally consistent with the DPPHsc data mentioned above (Figure 3A).

### 2.6. Anti-Inflammatory Activities of KSP in LPS-Stimulated RAW 264.7 Cells

**Figure 4:**
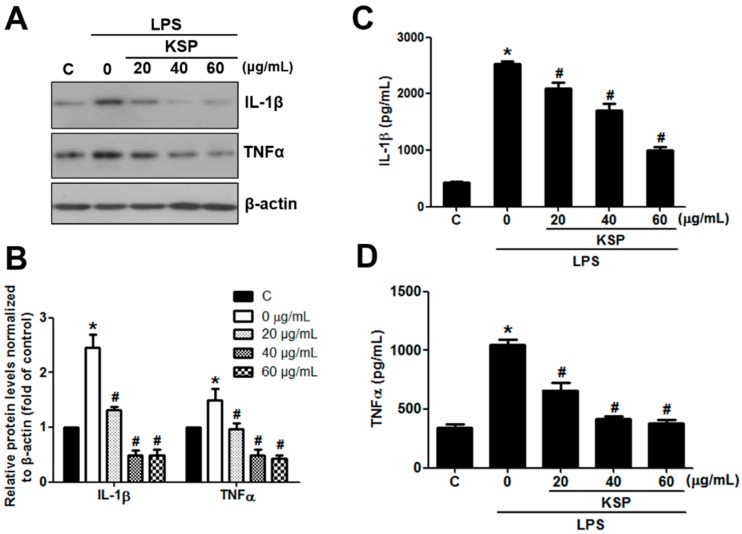
Secretion of IL-1β and TNFα inhibited by KSP in LPS-stimulated RAW 264.7 cells. (**A**,**B**) RAW 267.4 cells were pretreated with KSP (0, 20, 40, 60 μg/mL for 12 h) and then stimulated with LPS (1 μg/mL for 1 h) with KSP withdrawing. Protein was extracted and subjected to immunoblotting for IL-1β, TNFα and β-actin. Western blots were representative of three independent experiments; (**C**,**D**) The levels of IL-1β and TNFα were determined in the culture medium using ELISA kits. Data was analyzed by ANOVA followed by Student’s *t* test. Vertical bars represent the standard error from the means of three separate tests. * *p* < 0.05 vs. control, # *p* < 0.05 vs. LPS without KSP.

Inflammatory abnormalities are widely implicated in a large variety of acute and chronic human disease processes, leading to a wide range of pro-inflammatory cytokines and mediators such as interleukin-1β (IL-1β) and tumor necrosis factor-alpha (TNFα) [22]. To identify the effects of KSP on inflammation, we examined the expression and secretion levels of IL-1β and TNFα using Western blotting and ELISA kit analyses in LPS-induced RAW 264.7 cells. As compared to the control group, the group with LPS alone significantly induced the expression levels of IL-1β and TNFα (2.6- and 1.8-fold, respectively, *p* < 0.05). However, such an increase was found to be dramatically suppressed by the pretreatment with KSP for 12 h in a dose-dependent manner (Figure 4A,B). Compared with other groups, the expression levels of IL-1β and TNFα was almost lowered to the levels of the LPS group alone when we pretreated the cells with 60 μg/mL KSP (Figure 4A,B), demonstrating the elimination of LPS-induced inflammation in RAW 264.7 cells at higher KSP concentrations. Interestingly, we also observed the same inhibitory trend by measuring the levels of secreted IL-1β and TNFα in the culture medium, as detected by the ELISA kit (Figure 4C,D). Collectively, these results suggest that KSP can suppress the LPS-induced inflammation in RAW 264.7 cells. The production of reactive oxygen species has been known as a prerequisite for inflammation [23]. Therefore, utilizing antioxidants for reducing pro-inflammatory cytokines and mediators might be considered as an effective option for treating inflammation-related diseases. Previous studies have already demonstrated that natural antioxidants, such as procyanidins [24], resveratrol [25], and epigallocatechin-3-gallate [26] can inhibit inflammatory response through downregulated pro-inflammatory cytokines including caspase-1, IL-1β, and TNFα. Consistent with these reports, our study had also found that KSP, a natural polyphenol, exhibited a strong antioxidant activity (Figure 3A,B) that might be one of the ways to suppress the expression of IL-1β and TNFα. However, further studies are needed to establish a detailed relationship between the antioxidant of KSP and its anti-inflammatory activities.

### 2.7. Identification of Phenolic Compounds in KSP

**Figure 5:**
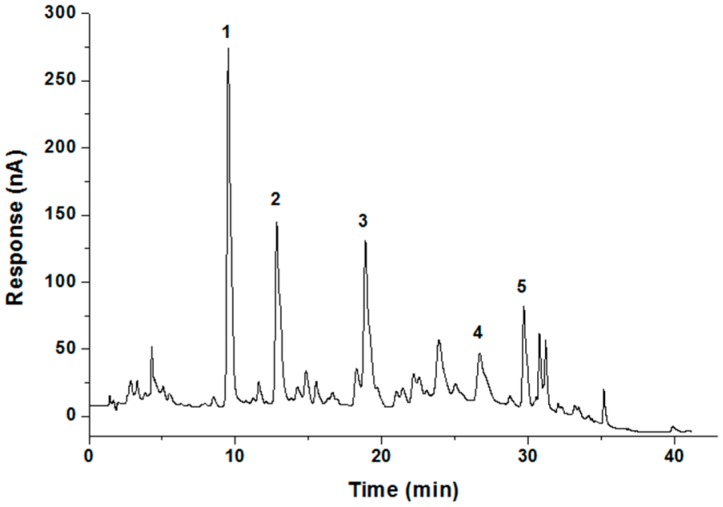
HPLC chromatograms of the KSP components (column, 150 mm × 4.6 mm, 5 μm; flow rate, 1.0 mL/min; eluent, methanol-water system; ECD voltage, 0.8 V). The peaks were identified as protocatechuic acid (1); *p*-hydroxybenzoic acid (2); caffeic acid (3); *p*-coumaric acid (4) and ferulic acid (5).

To identify the polyphenolic compounds present in the KSP, we performed an HPLC-ECD analysis (Figure 5), and the corresponding contents are summarized in Table 4. Peaks were identified by comparing the retention times (tR) with those of the reference standards. Peak 2 with a tR of 12.82 min, showing the maximum content (186.32 mg/g KSP) among the polyphenolic compounds, was identified as *p*-hydroxybenzoic acid. The second main peak (peak 1) with a tR of 9.51 min was identified as protocatechuic acid (96.80 mg/g KSP). The third main peak (peak 4) was identified as *p*-coumaric acid (81.65 mg/g KSP) with a tR of 26.69 min. The fourth and fifth main peaks were identified as caffeic acid (40.40 mg/g KSP) and ferulic acid (19.64 mg/g KSP), respectively. All the five main polyphenolic compounds account for 424.81 mg/g KSP and are all different from other plant resources, such as grade stem [27], apple pomace [28] and grape seeds [29]. The antioxidant and anti-inflammatory activities of KSP may be due to the synergetic effect of the five identified compounds and/or their combination to other extracted chemicals. Other studies reported that protocatechuic acid and *p*-hydroxybenzoic acid exhibit strong antioxidant, anticancer and antiatherogentic activities [30], whereas caffeic acid, *p*-coumaric acid, and ferulic acid have potential therapeutic effects in diabetes and hyperlipidemia, attributable to their strong antioxidant and anti-inflammatory properties [31]. However, in our study, whether the antioxidant and anti-inflammatory activities of KSP were directly related to these compounds or other undetected compounds needs to be further clarified in the future.

## 3. Materials and Methods

### 3.1. Materials

The following chemical compounds BHT, folin-ciocalteu reagent, DPPH, LPS, gallic acid, protocatechuic acid, *p*-hydroxybenzoic acid, caffeic acid, *p*-coumaric acid, ferulic acid, fetal bovine serum, IL-1β, and TNFα ELISA kits were purchased from Sigma-Aldrich (St. Louis, MO, USA). Rabbit polyclonal antibodies to IL-1β, TNFα, and β-actin were purchased from Abcam (Cambridge, UK). All other reagents used were of analytical grade or purer. Kiwi fruit seeds were obtained from Qinmei Co. (Xi’an, China). Grape seeds (*Cabernet sauvignon*) were supplied by Qinhuangdao Hwaseong Winery (Qinhuangdao, China). The raw materials were crushed into fine powder presenting a size range of 0.30–0.50 mm. The milled sample was defatted by a Soxhlet-extraction with *n*-hexane as the solvent. The defatted powder was placed at room temperature overnight to decant the residual *n*-hexane release and was then stored at −4 °C until used.

### 3.2. Polyphenols Extraction

The defatted sample (5 g) was homogenized in the extracting solvent with different levels of independent variables consisting of acetone concentration (50%–70% *v*/*v*), solid/liquid ratio (1:06–1:18), extraction time (70–90 min) and temperature (30–50 °C). The extraction conical flask was placed in a thermostatic water bath (HH-S2, Zhengji, China) and set at appropriate parameters, with shaking at a constant rate. The conical flask was covered with parafilm and aluminium foil to prevent solvent loss and light exposure during the extraction. Then the mixture was filtered and centrifuged at 12,000× *g* for 30 min (Auanti J-26XP, Beckman, Brea, CA, USA) to separate the insoluble materials. The supernatants were collected in an amber reagent bottle and stored at −20 °C until analysis. As the control sample, GSP was prepared by the same method described above with the same parameters as the KSP. All the experiments were carried out in triplicate. 

### 3.3. Experimental Design

The effects of four independent variables *x*_1_ (extraction time), *x*_2_ (acetone concentration), *x*_3_ (temperature) and *x*_4_ (solid/liquid ratio, *w*/*v*) at five variation levels on the TPC and the DPPHsc in the KSP extraction process were investigated using RSM. Each variable was coded at five levels: −2, −1, 0, 1, 2 (Table 1), according to the following equation:

(1) $$X_{i}=(x_{i}-x_{0})/\Delta x_{i}$$

where *X_i_* is the dimension value of an independent variable, *x_i_* is the real value of an independent variable, *x*_0_ is the real value of an independent variable at the center point, and Δ*x_i_* is the step change. The independent variables and their ranges were chosen based on preliminary experiment (the single factor test) results.

A central composite design (CCD) for RSM was arranged to fit the second-order model as shown in Table 2 [32]. The model proposed for the response (*Y*) was:

(2) $$Y=b_{0}+\sum_{n=1}^{4}b_{n}x_{n}+\sum_{n=1}^{4}b_{nn}{x_{n}}^{2}+\sum_{n<m}^{4}b_{mn}x_{n}x_{m}$$

where *b*_0_ is the value for the fitted response at the central point of the design, that is point (0,0,0,0). *b_n_*, *b_nn_,* and *b_mn_* are the linear, quadratic, and interaction regression coefficients, respectively. 

### 3.4. TPC Assay

The TPC was determined by the Folin-Ciocalteu colorimetric method [33]. Estimations were carried out in triplicate and calculated from a calibration curve obtained with gallic acid as the standard. The TPC was expressed as gallic acid equivalents in mg GAE/g DW.

### 3.5. DPPHsc Assay

The scavenging activity of the DPPH free-radical was estimated according to the method described by Ghasemi et al. [34]. Briefly, DPPH was dissolved in methanol to a final concentration (25 μg/mL). 5 mL of DPPH solution was mixed with the acetone extract (10–50 μg) and vibrated for 20 s. The absorbance of the samples and control was read at 517 nm after incubating at room temperature in the dark for 30 min. A methanolic solution of DPPH served to deduct the margin value. The scavenging percentage was calculated using the following equation:

DPPHsc (%) = (1 − ODs/ODc) × 100%
(3)

where ODs is the absorbance of DPPH radical + sample extract/standard; ODc is the absorbance of DPPH radical only.

### 3.6. FRAP Assay

The FRAP was determined according to that previously reported [35]. The acetone extract (10–50 μg) was dissolved in phosphate buffer (2.5 mL, 0.2 M, pH 6.6) and mixed with a potassium ferricyanide solution (2.5 mL, 1% *w*/*v*). The mixture was heated at 50 °C for 20 min. Then, trichloroacetic acid (5 mL, 10% *w*/*v*) was added to the mixture to quench the reaction. After centrifugation at 12,000× *g* for 10 min, the supernatant (2.5 mL) was moved into another tube containing a mixture of distilled water (2.5 mL) and ferric chloride (0.5 mL, 0.1% *w*/*v*). The absorbance of the sample and control was measured at 700 nm in triplicate after standing for 30 min at room temperature.

### 3.7. Determination of IL-1β and TNFα

RAW 264.7 cells were purchased from the Shanghai Institute of Biochemistry and Cell Biology and were cultured in DMEM medium supplemented with 10% fetal bovine serum. The cells were seeded into six-well plates and grown to 100% confluence at 37 °C in an atmosphere containing 5% CO_2_. The cells were then pretreated with different concentrations of KSP for 12 h and then stimulated with LPS (1 μg/mL) for 1 h. The levels of IL-1β and TNFα in the supernatants were determined using ELISA kits according to the manufacturer’s instructions. The levels of IL-1β and TNFα in the cells were determined using Western blotting described by Yang et al. [24].

### 3.8. HPLC-ECD Conditions

The separation of the KSP was performed with a Zorbax SB-C18 column (150 mm × 4.6 mm, 5 μm) (Agilent Technologies, Santa Clara, CA, USA) proceeded by a Zorbax SB-C18 guard column (20 mm × 4.0 mm, 5 μm) (Agilent Technologies). The mobile phase consisted of 0.5% (*v*/*v*) aqueous acetic acid (A) and methanol (B). The elution profile was as follows: 0–10 min, linear gradient 2%–15% B; 10–15 min, linear gradient of 15%–20% B; 15–25 min, linear gradient of 20%–25% B; 25–35 min, linear gradient of 25%–70% B. The samples were filtered through a Millipore 0.22 μm cellulose acetate filter. The flow rate was 1.0 mL/min, and 10 μL was injected into the HPLC system. Detection and quantification were carried out with the ECD operating at an applied voltage of 0.8 V. Identification was done by comparing the peak retention times with the ones of the standards.

### 3.9. Statistical Analyses

The data was analyzed by analysis of variance (ANOVA) and regression models using a statistical software Design-Expert 7.1.6 (Stat-Ease Inc., Minneapolis, MN, USA). A second-order polynomial was fitted to the main data to obtain the regression equations. The accuracy and general ability of the model could be evaluated by the coefficient of determination (R^2^). The statistical significance of the terms in the regression equations was examined. The surface plots were generated by assigning constant (zero) values to two of the four variables and solving the fitted equations as a quadratic equation in the remaining two variables. A *p* < 0.05 was considered significant. All experiments were triplicated.

## 4. Conclusions

In this study, we have successfully implemented RSM for optimizing the KSP extraction method, which ensures maximum recovery of TPC with high DPPH scavenging capacity. The KSP exhibited strong antioxidant and anti-inflammatory activities as indicated by the results of the DPPHsc, FARP and determination of IL-1β and TNFα in LPS-stimulated RAW 264.7 cells. Five polyphenolic compounds were identified in the KSP at optimized conditions using HPLC-ECD. On the basis of our findings discussed above, we can infer that this study has established an optimized method for extracting polyphenols from kiwi fruit seeds. The antioxidant and anti-inflammatory activities of KSP further emphasized the significance of an effective utilization of polyphenol rich by-products generated by kiwi fruit industries.

## Figures and Tables

**Table 1:** Independent variable values of the kiwi fruit seed polyphenols (KSP) extraction process and their corresponding levels.

Independent Variables	Symbol	Coded Variables Levels ^a^				
		−2	−1	0	1	2
Time (min)	*X*_1_	70	75	80	85	90
Acetone concentration (*v*/*v*)	*X*_2_	50	55	60	65	70
Temperature (°C)	*X*_3_	30	35	40	45	50
Solid/liquid ratio (*w*/*v*)	*X*_4_	1:06	1:09	1:12	1:15	1:18

^a^ Passage from coded variable (*Xi*) level to natural variable (*xi*) level is given by: *X*_1_ = 0.2*x*_1_ − 16; *X*_2_ = 0.2*x*_2_ − 12; *X_3_ =* 0.2*x*_3_ − 8; *X*_4_ = 1:(0.33*x*_4_ − 4).

**Table 2:** Central composite design (CCD) arrangement for the KSP extraction with respective coded factors, variable levels, responses and predicted values for total phenolic content (TPC) and DPPH scavenging capacities (DPPHsc) (number of samples *n* = 3).

Std	Run	Variables ^a^				TPC (mg GAE/g DW)		DPPHsc (%)	
		*x*_1_	*x*_2_	*x*_3_	*x*_4_	Experimental	Predicted	Experimental	Predicted
						*Y_e_*_1_	*Y_p_*_1_	*Y_e_*_2_	*Y_p_*_2_
27	1	80(0)	60(0)	40(0)	1:15(0)	53.73	49.99	62.75	61.77
28	2	80(0)	60(0)	40(0)	1:15(0)	48.49	49.99	64.86	61.77
9	3	75(−1)	55(−1)	35(−1)	1:18(1)	20.50	23.30	26.87	27.00
25	4	80(0)	60(0)	40(0)	1:15(0)	49.64	49.99	63.89	61.77
3	5	75(−1)	65(1)	35(−1)	1:12(−1)	34.83	34.64	43.65	44.52
8	6	85(1)	65(1)	45(1)	1:12(−1)	37.28	36.99	35.92	35.56
30	7	80(0)	60(0)	40(0)	1:15(0)	47.25	49.99	61.12	61.77
10	8	85(1)	55(−1)	35(−1)	1:18(1)	22.49	21.68	29.78	31.12
23	9	80(0)	60(0)	40(0)	1:09(−2)	55.10	49.72	48.21	51.43
7	10	75(−1)	65(1)	45(1)	1:12(−1)	39.12	37.19	49.26	42.64
16	11	85(1)	65(1)	45(1)	1:18(1)	35.39	33.98	42.62	41.28
6	12	85(1)	55(−1)	45(1)	1:12(−1)	40.48	39.91	36.65	36.56
26	13	80(0)	60(0)	40(0)	1:15(0)	52.28	49.99	61.29	61.77
22	14	80(0)	60(0)	50(2)	1:15(0)	37.02	41.16	43.32	47.71
14	15	85(1)	55(−1)	45(1)	1:15(1)	39.48	42.18	50.61	49.52
29	16	80(0)	60(0)	40(0)	1:15(0)	48.53	49.99	53.51	61.77
19	17	80(0)	50(−2)	40(0)	1:15(0)	37.76	37.69	36.13	36.97
31	18	80(0)	60(0)	40(0)	1:15(0)	50.13	49.99	64.96	61.77
11	19	75(−1)	65(1)	35(−1)	1:18(1)	21.94	19.78	27.83	22.64
17	20	70(−2)	60(0)	40(0)	1:15(0)	11.11	15.91	19.53	26.53
24	21	80(0)	60(0)	40(0)	1:21(2)	31.53	37.13	40.23	42.51
13	22	75(−1)	55(−1)	45(1)	1:18(1)	34.73	34.50	45.29	41.80
5	23	75(−1)	55(−1)	45(1)	1:12(−1)	36.06	40.10	46.27	45.80
1	24	75(−1)	55(−1)	35(−1)	1:12(−1)	34.21	32.88	45.58	41.64
20	25	80(0)	70(2)	40(0)	1:15(0)	30.90	31.26	26.89	31.61
15	26	75(−1)	65(1)	45(1)	1:18(1)	26.98	26.31	32.15	31.40
18	27	90(2)	60(0)	40(0)	1:15(0)	18.65	14.09	25.01	23.57
2	28	85(1)	55(−1)	35(−1)	1:12(−1)	22.42	23.38	28.27	28.80
21	29	80(0)	60(0)	30(−2)	1:15(0)	22.01	18.10	22.01	31.19
12	30	85(1)	65(1)	35(−1)	1:18(1)	19.67	18.15	28.63	28.92
4	31	85(1)	65(1)	35(−1)	1:12(−1)	27.65	25.14	35.62	33.84

^a^ *x*_1_: Time; *x*_2_: Acetone concentration; *x*_3_: Temperature; *x*_4_: Solid/liquid ratio.

**Table 3:** Polynomial equation and statistical parameters calculated after implementation of CCD design.

Regression Coefficient	Polynomial Equation	R^2^	Adjusted R^2^	*p* Value	Lack of Fit (*p* Value)
TPC (*Y*_1_)	50.01 + 0.48*X*_1_ − 0.88*X*_2_ + 4.83*X*_3_ − 4.08*X*_4_ − 0.14*X*_1_*X*_2_ + 2.19*X*_1_*X_3_* + 1.83*X*_1_*X*_4_ − 1.03*X*_2_*X*_3_ − 1.18*X*_2_*X*_4_ + 1.13*X*_3_*X*_4_ − 8.73*X*_1_^2^ − 3.87*X*_2_^2^ − 5.07*X*_3_^2^ − 1.62*X*_4_^2^	0.9654	0.9351	<0.0001	0.1764
DPPHsc (*Y*_2_)	61.77 − 0.74*X*_1_ − 1.34*X*_2_ + 4.13*X*_3_ − 2.23*X*_4_ + 0.54*X*_1_*X*_2_ + 0.90*X*_1_*X*_3_ + 4.24*X*_1_*X*_4_ − 1.51*X*_2_*X*_3_ − 1.81*X*_2_*X*_4_ + 2.66*X*_3_*X*_4_ − 9.18*X*_1_^2^ − 6.87*X*_2_^2^ − 5.58*X*_3_^2^ − 3.70*X*_4_^2^	0.9420	0.8913	<0.0001	0.3545

**Table 4:** Determination of the five polyphenolic compounds in the kiwifruit seeds ^a^.

Compounds	Retention Time (tR) (min)	Amount Detected (mg·g^−1^ KSP)
protocatechuic acid	9.51	96.80 ± 1.93
*p*-hydroxybenzoic acid	12.82	186.32 ± 3.05
caffeic acid	18.89	40.40 ± 1.73
*p*-coumaric acid	26.69	81.65 ± 2.25
ferulic acid	29.71	19.64 ± 1.12

^a^ Values are the means ± standard deviations (*n* = 3).

## Abbreviations

ANOVA	Analysis of Variance
BHT	Butylated Hydroxytoluene
CCD	Central Composite Design
DPPH	2,2-Diphenyl-1-Picrylhyclrazyl Radical
FRAP	Ferric Reducing Antioxidant Power
GAE	Gallic Acid Equivalents
GSP	Grape Seed Polyphenols
HPLC-ECD	High Performance Liquid Chromatography-Electrochemical Detector
IL-1β	Interleukin-1β
KSP	Kiwi Fruit Seed Polyphenols
LPS	Lipopolysaccharides
RSM	Response Surface Methodology
TPC	Total Phenolic Content
TNFα	Tumor Necrosis Factor-Alpha
